# Decomposition-reconstruction-optimization framework for hog price forecasting: Integrating STL, PCA, and BWO-optimized BiLSTM

**DOI:** 10.1371/journal.pone.0324646

**Published:** 2025-06-27

**Authors:** Xiangjuan Liu, Yunlong Li, Fengtong Wang, Yujie Qin, Zhongyu Lyu

**Affiliations:** 1 College of Computer and Control Engineering, Qiqihar University, Qiqihar, China; 2 Heilongjiang Key Laboratory of Big Data Network Security Detection and Analysis, Qiqihar University, Qiqihar, China; 3 College of Mechanical and Electrical Engineering, Fujian Agriculture and Forestry University, Fuzhou, China; Czestochowa University of Technology: Politechnika Czestochowska, POLAND

## Abstract

This study constructs a multi-stage hybrid forecasting model using hog price time series data and its influencing factors to improve prediction accuracy. First, seven benchmark models including Prophet, ARIMA, and LSTM were applied to raw price series, where results demonstrated that deep learning models significantly outperformed traditional methods. Subsequently, STL decomposition decoupled the series into trend, seasonal, and residual components for component-specific modeling, achieving a 22.6% reduction in average MAE compared to raw data modeling. Further integration of Spearman correlation analysis and PCA dimensionality reduction created multidimensional feature sets, revealing substantial accuracy improvements: The BiLSTM model achieved an 83.6% cumulative MAE reduction from 1.65 (raw data) to 0.27 (STL-PCA), while traditional models like Prophet showed an 82.2% MAE decrease after feature engineering optimization. Finally, the Beluga Whale Optimization (BWO)-tuned STL-PCA-BWO-BiLSTM hybrid model delivered optimal performance on test sets (RMSE = 0.22, MAE = 0.16, MAPE = 0.99%, ${\text{R}}^{2}\;=\;0.98$), exhibiting 40.7% higher accuracy than unoptimized BiLSTM (MAE = 0.27). The research demonstrates that the synergy of temporal decomposition, feature dimensionality reduction, and intelligent optimization reduces hog price prediction errors by over 80%, with STL-PCA feature engineering contributing 67.4% of the improvement. This work establishes an innovative “decomposition-reconstruction-optimization” framework for agricultural economic time series forecasting.

## 1 Introduction

In the context of global economic integration, the stability and prosperity of the agricultural products market are directly related to the healthy development of national economies and the well-being of society and people’s livelihoods. Among them, the hog industry not only bears the important mission of meeting residents’ basic living needs but is also one of the pillar industries of China’s agricultural economy. Pork is the most consumed meat globally on a per capita basis [1]. Pork, as the mainstay of China’s meat consumption structure, its price fluctuations not only directly affect consumers’ living costs but also deeply impact hog farmers’ economic interests, feed production enterprises’ sales strategies, and the resource allocation efficiency throughout the entire industrial chain [2]. Excessive price fluctuations can not only impact producers’ and consumers’ cost expenditures but also complicate government policy regulation [3]. Especially after significant outbreaks such as the African swine fever, the complexity and uncertainty of the hog market have significantly increased, with pork price fluctuations exhibiting more pronounced non-stationarity and nonlinear characteristics, posing a significant challenge to traditional forecasting methods. Therefore, the construction of more accurate and efficient hog price forecasting models has become a common focus of attention for academia and industry.

Scholars from various countries have conducted extensive and in-depth research on agricultural product price forecasting, but there are still many issues worth exploring in the specific field of hog price forecasting. On the one hand, pig prices are jointly influenced by multiple factors such as feed costs, breeding technology, market demand, and epidemic prevention and control. The intricate interactions among these factors are difficult to quantify simply. On the other hand, factors such as economic policy uncertainty and information asymmetry in the live pig market exhibit time variability and distinct characteristics, and the influence of external shocks on industry pricing demonstrates a patterned trend in line with historical tendencies, further complicating price forecasting [4]. Therefore, leveraging modern statistical analysis methods, data mining techniques, and machine learning algorithms to deeply explore the underlying patterns and trends in hog prices holds significant theoretical and practical importance [5].

This study innovatively proposes a comprehensive forecasting model, which ingeniously integrates the temporal characteristics of price data with relevant influencing factor data, successfully overcoming the limitations of previous models in the application of temporal features. Addressing the common challenges in hog price forecasting, such as the lag in prediction results and the difficulty in accurately fitting “jumpy” data, this study adopts a series of strategies including data decomposition, constructing an appropriate model, data dimensionality reduction, and intelligent optimization to effectively tackle these issues. Through these measures, the model has achieved remarkable results in enhancing the comprehensiveness and accuracy of the analysis process.

## 2 Review of literature

Currently, the forecasting methods adopted by scholars from various countries in the field of agricultural product price forecasting can be roughly divided into Traditional Time Series Forecasting Methods, Machine learning prediction methods and combined forecasting methods.

Traditional Time Series Forecasting Methods utilize historical data and statistical methodologies to predict agricultural product prices. Some commonly used Traditional Time Series Forecasting Methods include methods such as Exponential Smoothing [6], Autoregressive Moving Average (ARMA) [7], Autoregressive Integrated Moving Average (ARIMA) [8], Autoregressive Conditional Heteroskedasticity (ARCH) [9], Grey Forecasting [10], and Holt-Winters [11].

For example, in 2020, Mehmet Arif Şahinli [12] accurately predicted the upcoming four-month price trend of potatoes in Turkey utilizing the Holt-Winters and ARIMA(1,1,2) models. In 2024, Yiyang Qiao *et al*. [13] initially processed the price data of Korean green onions using the Christiano-Fitzgerald filter and the CensusX-13 seasonal adjustment methods. Following this, they constructed an ARMA(1,2)-GARCH(1,1) model to forecast the price data of Korean green onions for the subsequent eight months. In 2019, Wilfrido Jacobo Paredes-Garcia *et al*. [14] established a SARIMA model to anticipate the prices of fruits and vegetables in the Queretaro state. In 2024, Jan BanaÂ´s *et al*. [15] employed ARIMA, SARIMA, and SARIMAX models to predict the nominal prices of pine, spruce, beech, birch, and alder roundwood, as well as the construction confidence index (CCI), incorporating the CCI lagged by three quarters as an influencing factor. Additionally, in 2024, Kumar, Vibhanshu *et al*. [16] combined the GARCH and Holt-Winters models to forecast the seasonality and volatility in three distinct agro-climatic zones of India. Traditional time series forecasting methods primarily rely on the patterns of historical data to construct predictive models. This approach is particularly effective when the series exhibits clear autocorrelation and stability, excelling in addressing prediction problems with obvious linear relationships. However, traditional methods struggle to cope with the prevalent nonlinear characteristics found in complex systems such as price series, which can lead to inaccurate prediction results. Therefore, traditional time series forecasting methods need to provide satisfactory forecasting performance.

With the advent and rapid development of artificial intelligence, various machine learning methods have become the mainstream for predicting agricultural product prices. These include Recurrent Neural Networks (RNNs) [17], Gated Recurrent Units (GRUs) [18], Long Short-Term Memory (LSTM) neural networks [19], Support Vector Machines (SVMs) [20], Random Forests (RFs) [21], and others.

For example, in 2020, Tserenpurev Chuluunsaikhan *et al*. [22] utilized the LDA model to extract thematic information from 10,854 online news articles for feature selection, and combined this with data from 2010 to 2018 to construct an LSTM model for predicting hog prices in 2019. In 2019, Xiaoquan Chu *et al*. [23] proposed the combined model EEMD-ADD, which involves processing grape price data with EEMD and then combining Support Vector Regression (SVR) and Multiple Linear Regression (MLR) to predict the trend of grape price in China. In 2022, Lianlian Fu *et al*. [24] combined Ensemble Empirical Mode Decomposition (EEMD) with Multi-Long Short-Term Memory Neural Networks (Multi-LSTMs) to forecast future hog prices, and performed comparative experiments with LightGBM and MLP. In 2022, Chang Xu *et al*. [25] introduced a method based on Lasso and RAsy-v-TSVR to predict soybean prices in China, where RAsy-v-TSVR is an improvement of Asymme- tric v-twin Support Vector Regression (Asy-v-TSVR), addressing the potential issue of matrix irreversibility in Asy-v-TSVR. In 2015, Tao Xiong *et al*. [26] extended this approach to forecast interval-valued agricultural commodity futures prices using the Vector Error Correction Model (VECM) and Multi-Output Support Vector Regression (MSVR) (abbreviated as VECM-MSVR), which is capable of capturing linear and non-linear patterns exhibited in agricultural commodity futures prices. In 2022, Cerqueira, V. *et al*. [27] mentioned that some evidence suggested that machine learning methods performed poorly in terms of predictive performance compared to simpler statistical methods. However, Cerqueira, V., and his team refuted this viewpoint, arguing that statistical methods were only effective in cases with extremely limited sample sizes. By applying the learning curve methodology, they demonstrated how machine learning methods improved their relative predictive capabilities as the sample size increased. In 2019, Li *et al*. [28] utilized the Transformer architecture to address time series problems and proposed the LogSparse Transformer model to tackle the two major issues of local uncertainty and memory bottlenecks inherent in the Transformer. This model boasts low memory overhead, fine granularity, and strong long-term correlation capabilities, enabling precise predictions of time series data for both synthetic and real-world datasets.

However, there are also some drawbacks and challenges. For instance, machine learning models often encounter limitations in comprehensively and accurately capturing the intricate and dynamic features and patterns within time series data due to their inherent mechanisms, and can encounter problems such as overfitting and local optimization, which inevitably leads to biases or constraints in prediction results.

To overcome this challenge, combined forecasting systems have emerged as an innovative solution. These models intelligently integrate the strengths of different prediction techniques through careful design and fusion, organically combining multiple individual models to ensure that each model can fully leverage its expertise within its domain. Through complementarity and synergy, ensemble forecasting models not only enhance the robustness of the prediction system, but also improve the stability and reliability of prediction results, making them a significant development direction in the current field of time series forecasting [29]. In 2022, Junhao Wu *et al*. [30] introduced VMD-IBES-LSTM, a hybrid model integrating Variational Modal Decomposition, Improved Bald Eagle Search Algorithm, and LSTM, to forecast prices of five aquatic products. They discovered that compared to traditional and “feature extraction-prediction” models, their “decomposition-prediction-integration” methodology significantly bolstered robustness and precision. In 2020, Luyao Wang *et al*. [31] mentioned that prediction models for agricultural product prices have evolved from qualitative to quantitative, traditional to intelligent prediction models, and from single to hybrid models. In 2022, The research conducted by Benchimol, J., *et al*. [32] proposes a methodology for evaluating prediction performance under market turbulence conditions, which holds particular relevance for discussing model performance in the hog industry during periods of high market volatility. In 2023, Abdullan *et al*. [33] combined ARIMA with Artificial Neural Networks (ANN), using the ARIMA-NARNET model to forecast coconut prices. In 2021, Keqiang Li *et al*. [34] employed actual signal energy (AE-VMD) and a multi-scale adaptive Lempel-Ziv complexity calculation method (MA-LZ) to process pork, beef, and mutton price data, followed by predictions using a heterogeneous GRU neural network (AH-GRU). In 2018, Baojia Wang *et al*. [35] noted that since the ARIMA model cannot handle nonlinear data, they combined ARIMA with SVM and tested the predictive effectiveness of ARIMA-SVM on garlic prices from 2010-2017, finding that the combination of ARIMA and SVM achieved better prediction results. In 2018, Rangapuram, S.S. *et al*. [36], proposed a novel method for probabilistic time series forecasting that integrates state space models with deep learning. By parametrizing the linear state space model for each time series using a jointly learned recurrent neural network, this method can scale from scenarios with limited training data to scenarios where millions of time series data can be utilized to learn accurate models. In 2023, Barkan, O., *et al*. [37] proposed a hierarchical architecture based on recurrent neural networks, known as the Hierarchical Recurrent Neural Network (HRNN) model, for predicting disaggregated inflation components within the Consumer Price Index (CPI). This model utilizes information from higher levels within the CPI hierarchy to improve predictions of the more volatile lower-level inflation components. Evaluations demonstrate that the HRNN model significantly outperforms a range of well-known inflation prediction baseline models in terms of forecasting performance. Therefore, these hybrid models under study can fully exploit the features and advantages of different models to improve the completeness of forecasts.

In exploring the diverse methodologies within the realm of time series forecasting, some scholars tend to adopt time series decomposition techniques or digital signal processing methods to preprocess time series data. These approaches encompass, but are not limited to, Empirical Mode Decomposition (EMD) [38], Variational Mode Decomposition (VMD) [39], Complete Ensemble Empirical Mode Decomposition with Adaptive Noise (CEEMDAN) [40], Singular Spectrum Decomposition (SSD) [41], and Seasonal and Trend Decomposition using Loess (STL) [42]. These decomposition techniques effectively break down complex time series data into more manageable and analyzable components such as trend, seasonal, and random noise, thereby offering a richer perspective and strategic options for time series forecasting. Through the application of these methods, scholars can gain a deeper understanding of the intrinsic structure and characteristics of time series data, ultimately facilitating the design of more precise and efficient prediction models.

In summary, agricultural product price forecasting methods encompass traditional time series approaches as well as intelligent prediction techniques such as machine learning and artificial neural networks. In terms of prediction accuracy, neural network models demonstrate improved precision compared to conventional statistical models. However, each model inherently possesses limitations, and combined models—unlike single models—can integrate the strengths of different methodologies to further enhance forecasting accuracy. Based on this rationale, the present study integrates STL (Seasonal-Trend decomposition using Loess), SARIMA (Seasonal AutoRegressive Integrated Moving Average), PCA (Principal Component Analysis), BWO (Beluga Whale Optimization) algorithm, and Bidirectional Long Short-Term Memory (BiLSTM) neural networks. Through the fusion of these techniques, we aim to significantly improve the accuracy and stability of agricultural price predictions.

The STL model exhibits robust stability against outliers and is employed for preliminary decomposition of time series data into trend, seasonal, and residual components. The SARIMA model excels in forecasting cyclical time series patterns and is utilized to train seasonal components. LSTM networks adapt rapidly to abrupt changes in time series data and possess long-term memory capabilities, making them suitable for training residual and trend components. PCA, a statistical dimensionality reduction technique, simplifies the analysis of multifactor variables influencing pig prices. BiLSTM retains LSTM’s advantages while its bidirectional structure enables data memorization from both past and future directions, thereby enhancing prediction precision. The incorporation of the BWO optimization algorithm optimizes BiLSTM model parameters, ultimately generating refined output to achieve precise pig price forecasting.

## 3 Materials and methods

### 3.1 Dataset description

This study aims to explore effective avenues for agricultural product forecasting by predicting hog prices in China. The data used in the research comprises time series data on hog prices and data on influencing factors. Specifically, the hog price, corn price, and soybean meal price data were sourced from China Hog Net, while the remaining data were obtained from the Ministry of Agriculture and Rural Affairs of China. The data covers a time span from January 1, 2016, to May 19, 2024. For experimental convenience, this period is grouped by weeks, and the corresponding weekly data are derived by calculating averages. As such, the time span is represented as Week 1, 2016, to Week 20, 2024, totaling 418 weeks. In this experiment, the data from the first 318 weeks were used as the training set, and the subsequent 100 weeks were used as the test set.

This study utilizes libraries such as pandas, scikit-learn, seaborn, and statsmodels in Python for data cleaning and statistical analysis.

Addressing the issue of missing price data due to factors such as holidays, given that agricultural product price data often exhibit complex trends and seasonal patterns, this study selects cubic spline interpolation as the strategy for data imputation. Cubic spline interpolation is an effective mathematical tool that reasonably estimates the values of missing data points by considering the relationships among known data points, thereby maintaining data continuity and integrity. The mathematical expression of cubic spline interpolation is based on constructing a series of smoothly connected cubic polynomial functions, each defined over the interval between adjacent data points. For the i-th interval $\left[x_{i},x_{i+1}\right]$, the cubic spline function *S*_*i*_(*x*) is given by Eq 1:

$$S_{i}\left(x\right)=a_{i}+b_{i}\left(x-x_{i}\right)+c_{i}{\left(x-x_{i}\right)}^{2}+d_{i}{\left(x-x_{i}\right)}^{3}$$ (1)

In the Eq 1, *x* is the point where interpolation is needed, *x*_*i*_ and *x*_*i* + 1_ are the endpoints of the interval, and *a*_*i*_, *b*_*i*_, *c*_*i*_, and *d*_*i*_ are the polynomial coefficients. More detailed information is provided in Table 1.

**Table 1:** Descriptive statistical analysis of data.

Variables	Min	Max	Mean	Std	Kurtosis	Skewness
Live pig	10.23	37.82	19.04	7.16	0.21	1.22
Corn	1.58	2.96	2.24	0.47	-1.64	0.25
Soybean meal	2.55	5.69	3.57	0.67	0.46	1.09
White radish	1.02	3.23	1.65	0.33	2.52	1.18
White-striped pork	15.79	52.47	26.39	9.44	0.35	1.3
Beef	52.38	79.29	66.31	9.71	-1.65	-0.18
Dressed chicken	12.8	20.94	16.47	1.68	-0.74	-0.05
Large yellow croaker	39.72	48.17	43.03	1.43	-0.01	0.09
Agricultural Product Wholesale Price 200 Index	93.07	142.24	115.46	11.87	-0.94	-0.09
Import volume of live pigs	32820.81	143207.6	69000.14	27487.43	0.41	1.18
Export volume of live pigs	1548.75	10363.69	5028.64	1861.4	0.09	0.59
Import value of live pigs	5889.01	39460.99	15613.76	8754.1	0.28	1.19
Export value of live pigs	738.4	3580.1	1954.98	539.49	0.1	0.24

**Fig 1:**
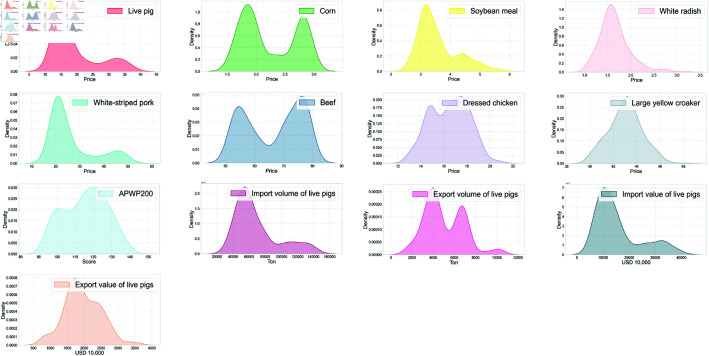
The kernel density plot for data of each feature.

Table 1 indicates that the kurtosis values of Live pig and its various influencing factors are generally low. This characteristic indicates that their distribution patterns are relatively flat, with data points scattered more widely around the mean and exhibiting a lower concentration. Furthermore, the data for Live pig exhibits a right-skewed trend, which implies that in the distribution of price data, data points are more concentrated on the lower value side, while those on the higher value side are relatively sparse. This distribution pattern suggests a tendency for live pig prices to fluctuate upwards. Fig 1 displays the kernel density for each feature. The kernel density plot generates a continuous probability density function by smoothing the data points, allowing for a more intuitive visualization of the distribution of each feature.

### 3.2 STL

The Seasonal-Trend decomposition procedure using Loess (STL), introduced by Cleveland [43] in 1990, employs LOESS (Locally Weighted Scatterplot Smoothing) technique to estimate the trend $T_{V}$, seasonal $S_{V}$, and residual components $R_{V}$ in a time series through a series of smoothing processes, as depicted in Eq 2.

$$Y_{V}=T_{V}+S_{V}+R_{V}$$ (2)

**Fig 2:**
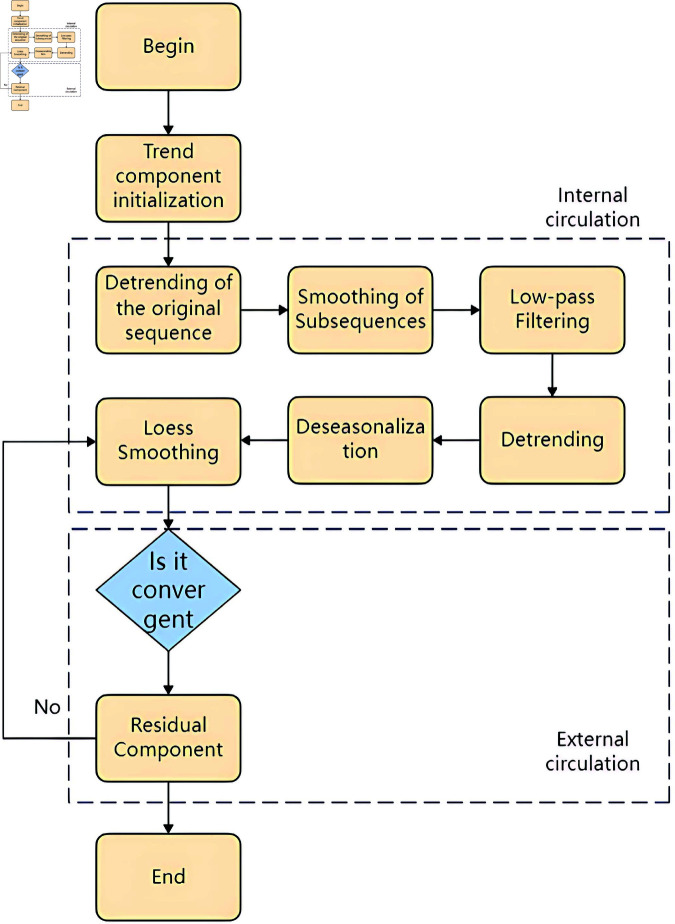
Flowchart of the STL.

The STL primarily consists of two recursive processes: an inner loop nested within an outer loop, as illustrated in Fig 2.

During each iteration of the inner loop, the seasonal and trend components are updated once. Each iteration of the outer loop recalculates the weights, which are then used for the iterations of the inner loop. By default, the weights for the initial loop are all set to 1. In this experiment, STL (Seasonal and Trend decomposition using Loess) is utilized to decompose the raw data into three components: the trend component, the seasonal component, and the residual component.

### 3.3 PCA

PCA (Principal Component Analysis) [44] is a commonly used method for representing high-dimensional data with low-dimensional data. Extracting principal components can not only eliminate the correlation between sample data but also reduce the computational load of the model and improve its prediction accuracy. Suppose the original variable vector is $X=[x_{1},x_{2},...,x_{n}]$, where *x*_1_,*x*_2_,...,*x*_*n*_ are the original variables and n is the dimensionality of the variables. After dimensionality reduction, a new variable vector $Y=[y_{1},y_{2},...,y_{m}]$ can be obtained, where *y*_1_,*y*_2_,...,*y*_*m*_ are the newly generated variables, m is the dimensionality of the new variables, and *m* < *n*. The formula for calculating y m is:

$$\left\{ \begin{array}{c} y_{1}=e_{11}x_{1}+e_{12}x_{2}+...+e_{1n}x_{n}\\ y_{2}=e_{21}x_{1}+e_{22}x_{2}+...+e_{2n}x_{n}\\ ...\\ y_{m}=e_{k1}x_{1}+e_{k2}x_{2}+...+e_{kn}x_{n} \end{array}\right.$$ (3)

### 3.4 BWO

With the rise of artificial intelligence, metaheuristic algorithms have been widely applied to optimization problems in recent years. The Beluga Whale Optimization (BWO) algorithm is a population-based metaheuristic algorithm proposed by ZHONG *et al*. [45] in 2022. It simulates the behaviors of beluga whale pods such as paired swimming, centralized predation, and whale-fall to find optimal solutions within the solution space.

The BWO algorithm transitions from global exploration to local exploitation based on the balance factor *B*_*f*_. When the balance factor *B*_*f*_ > 0.5, it is in the global exploration phase; when the balance factor *B*_*f*_ < 0.5, it is in the local exploitation phase. The balance factor B can be expressed as:

$$B_{f}=B_{0}\cdot \left[1-\frac{t}{2T}\right]$$ (4)

In the Eq 4: t represents the current iteration number; T represents the maximum number of iterations; *B*_0_ is a random number within (0, 1).

The inspiration for the global exploration phase of the BWO algorithm comes from the paired swimming behavior of beluga whales. The expression for the position $x_{i,j}^{t+1}$ of the i-th individual beluga whale in the j-th dimension is:

$$\left\{ \begin{array}{c} x_{i,j}^{t+1}=x_{i,p}^{t}+\left(x_{r,p}^{t}-x_{i,p}^{t}\right)\left(1+r_{1}\right)\sin\left(2\pi r_{2}\right),j=2n\\ x_{i,j}^{t+1}=x_{i,p}^{t}+\left(x_{r,p}^{t}-x_{i,p}^{t}\right)\left(1+r_{1}\right)\cos\left(2\pi r_{2}\right),j=2n+1 \end{array}\right.$$ (5)

In the Eq 5: both *r*_1_ and *r*_2_ are random numbers between (0, 1); $x_{i,p}^{t}$ represents the position of the i-th individual on a randomly selected dimension p at the current iteration; and $x_{r,p}^{t}$ represents the position of a randomly selected individual r on the same randomly selected dimension p at the current iteration.

The inspiration for the local exploitation phase of the BWO algorithm comes from the predation behavior of beluga whales. The expression for the updated position $x_{i}^{t+1}$ of the i-th beluga whale, given its original position $x_{i}^{t}$, is:

$$x_{i}^{t+1}=r_{3}\cdot x_{best}^{t}=r_{4}\cdot x_{i}^{t}+C_{1}\cdot L_{F}\cdot \left(x_{r}^{t}-x_{i}^{t}\right)$$ (6)

In the Eq 6: both *r*_3_ and *r*_4_ are random numbers between (0, 1); $C_{1}=2\cdot r_{4}\cdot (1\;-\;t/T)$ represents the random jump degree, which measures the intensity of Lévy flight; $x_{best}^{t}$ is the current optimal position of the beluga whale; $x_{r}^{t}$ is the position of a randomly selected individual r from the current population; $x_{i}^{t}$ is the position of the i-th individual at the current iteration; *L*_*F*_ is a random number following a Lévy distribution, and its expression is:

$$L_{F}=0.05\times \mu /|v{|}^{1/\beta }\times {\left[\frac{𝛤\left(1+\beta \right)\times \sin\left(\pi \cdot \beta /2\right)}{𝛤\left(\left(1+\beta \right)/2\right)\times \beta \times 2^{\left(\beta -1\right)/2}}\right]}^{1/\beta }$$ (7)

In the Eq 7: β=1.5; both μ and *v* are random numbers following a normal distribution, i.e., μ,υ N(0,1); 𝛤(1+β) represents the 𝛤 function, which is defined as 𝛤(x)=(x−1)!.

Assuming that belugas either migrate to other places or experience whale falls and sink into the deep sea, in order to maintain the population size, a position update formula is established using the current position of the beluga and the step size of the whale fall. The expression for the updated position $x_{i}^{t+1}$ of the i-th individual beluga with initial position $x_{i}^{t}$ is:

$$\left\{ \begin{array}{c} x_{i}^{t}=r_{5}\cdot x_{i}^{t}-r_{6}\cdot x_{r}^{t}+r_{7}\cdot x_{step}^{t}\\ x_{step}^{t}=e^{-C_{2}\cdot t/T}\cdot \left(U_{b}-L_{b}\right) \end{array}\right.$$ (8)

In the Eq 8: *r*_5_, *r*_6_, and *r*_7_ are all random numbers between (0, 1); *L*_*b*_ and *U*_*b*_ represent the lower and upper bounds of the optimization problem, respectively; $x_{step}^{t}$ denotes the step length of whale fall; $C_{2}=2W_{f}\;\times \;N$ represents the step factor; *W*_*f*_ represents the probability of whale fall, and $W_{f}=0.1\;-\;0.05t/T$ is a linear function where the probability of whale fall decreases from 0.1 to 0.05. This indicates that as the beluga whales get closer to the food source during the optimization process, their risk decreases, which also implies a closer proximity to the optimal solution.

### 3.5 SARIMA

Seasonal Autoregressive Integrated Moving Average (SARIMA) is an extension of the Autoregressive Integrated Moving Average (ARIMA) model and is one of the most prevalent models in time series forecasting. The basic idea of the ARIMA model is to treat the data series of the prediction target over time as a stochastic sequence and use a mathematical model to roughly describe or simulate this sequence. Once the ARIMA model is identified, it can predict future values of the time series based on the relationship between past and present values.

In the ARIMA model, there are three main components: Autoregressive (AR), Integrated (I), and Moving Average (MA). The ARIMA model can be represented as ARIMA(p,d,q), where:

- p represents the number of non-seasonal autoregressive terms.
- d represents the number of non-seasonal differences.
- q represents the number of non-seasonal moving average terms.

The (p,d,q) notation refers to the non-seasonal part of the model.

The SARIMA (Seasonal Autoregressive Integrated Moving Average) forecasting model can be represented as ARIMA(p,d,q)(P,D,Q)s, which can be understood as the ARIMA(p,d,q) model with the addition of the seasonal period (s). Here:

- P represents the seasonal autoregressive terms.
- D represents the seasonal differences.
- Q represents the seasonal moving average terms.

This is shown in Eqs 9 and 10.

$$\varphi_{p}\left(B\right)\phi_{P}\left(B^{S}\right)\nabla^{d}\nabla_{S}^{D}Z_{t}=\theta_{q}\left(B\right){𝛩}_{Q}\left(B^{S}\right)\alpha_{t}$$ (9)

$$\left\{ \begin{array}{c} \varphi_{p}\left(B\right)=1-\sum_{i_{p}=1}^{p}\varphi_{i_{p}}B^{i_{p}}\\ \phi_{P}\left(B\right)=1-\sum_{i_{p}=1}^{p}\phi_{i_{P^{S}}}B^{i_{P^{S}}}\\ \theta_{q}\left(B\right)=1-\sum_{i_{q}=1}^{q}\theta_{i_{q}}B^{i_{q}}\\ {𝛩}_{Q}\left(B^{S}\right)=1-\sum_{i_{Q}}^{Q}{𝛩}_{i_{Q^{S}}}B^{i_{Q^{S}}} \end{array}\right.$$ (10)

In the above equations:

- $\varphi_{p}$ represents the autoregressive operator for p.
- $\phi_{P}$ represents the seasonal autoregressive operator for P.
- $\nabla^{d}$ represents the differencing operator.
- $\nabla_{S}^{D}$ represents the seasonal differencing operator.
- *Z*_*t*_ represents the observed value at time point t.
- ${\text{B}}^{\text{S}}$ denotes the seasonal differencing operation.
- $\theta_{q}$ is the moving average operator for q.
- ${𝛩}_{Q}$ is the seasonal moving average operator for Q.
- $\alpha_{t}$ represents the white noise component of the stochastic model.

In this experiment, SARIMA is used to establish an appropriate model for the seasonal component and to forecast the seasonal component.

### 3.6 BiLSTM

**Fig 3:**
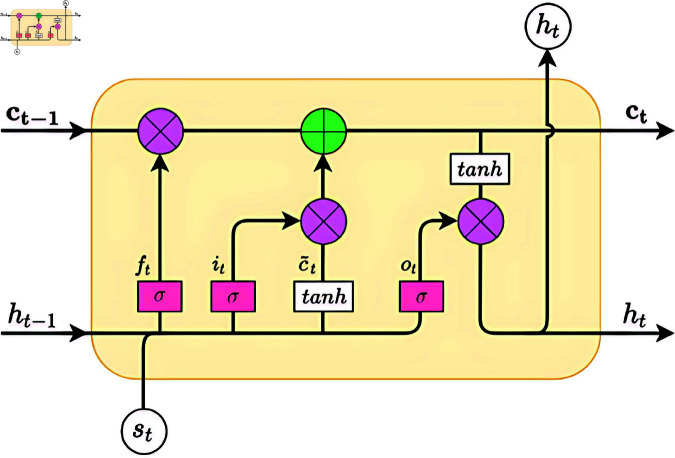
Internal structure of an LSTM cell.

Long Short-Term Memory (LSTM) is a special type of Recurrent Neural Network (RNN) that introduces a structure known as “memory cells” to address the vanishing and exploding gradient problems that arise during the training of long sequences. Each LSTM unit comprises an input gate *i*_*t*_, a forget gate *f*_*t*_, an output gate *o*_*t*_, a candidate cell state ${\tilde{c}}_{t}$, a cell state *c*_*t*_, and a hidden state *h*_*t*_, as illustrated in Fig 3. The input gate *i*_*t*_ determines whether the current input information is written into the cell state *c*_*t*_. The forget gate *f*_*t*_ decides if the information in the cell state is to be forgotten. The output gate *o*_*t*_ determines whether the information in the memory cell is outputted.

The input gate determines which new information will be added to the cell state *c*_*t*_. It also consists of two parts: a Sigmoid layer that decides which values will be updated, and a tanh layer that generates a new candidate value vector ${\tilde{c}}_{t}$.

$$i_{t}=\sigma \left(W^{\left(i\right)}\cdot \left(h_{t-1}⊕x_{t}\right)+b^{\left(i\right)}\right)$$ (11)

$${\tilde{c}}_{t}=\tan\text{h}\left(W^{\left(i\right)}\cdot \left(h_{t-1}⊕x_{t}\right)+b^{\left(c\right)}\right)$$ (12)

The forget gate determines which information to discard from the previous cell state $c_{t}\;-\;1$. It consists of a Sigmoid function, with inputs being the current time step’s input *x*_*t*_ and the output of the previous LSTM unit *h*_*t*_−1. The output of the Sigmoid function is a value between 0 and 1, which controls the degree of information retention (0 indicates complete forgetting, while 1 indicates complete retention).

$$f_{t}=\sigma \left(W^{\left(f\right)}\cdot \left(h_{t-1}⊕x_{t}\right)+b^{\left(f\right)}\right)$$ (13)

The update of the cell state *c*_*t*_ is achieved by combining the results of the forget gate and the input gate. First, the result of the forget gate *f*_*t*_ is multiplied by the old cell state $c_{t}\;-\;1$ to forget the unnecessary information. Then, the result of the input gate *i*_*t*_ is multiplied by the new candidate value vector ${\tilde{c}}_{t}$ to add new information.

$$c_{t}=f_{t}\times c_{t-1}+i_{t}+{\tilde{c}}_{t}$$ (14)

The output gate determines the output *h*_*t*_ for the current time step. It first passes through a Sigmoid layer to decide which information from the cell state will be output. Then, the cell state *c*_*t*_ is processed through a tanh function and multiplied by the output of the Sigmoid layer to obtain the final output *h*_*t*_.

$$o_{t}=\sigma \left(W^{\left(o\right)}\cdot \left(h_{t-1}⊕x_{t}\right)+b^{\left(o\right)}\right)$$ (15)

$$h_{t}=o_{t}\times \tan\text{h}\left(c_{t}\right)$$ (16)

In formula (11-16), σ represents the sigmoid function, ⊕ denotes the concatenation operator, and + and × symbolize element-wise addition and multiplication operations, respectively. $W^{\left(x\right)}$ and $b^{\left(x\right)}$ are the weight matrix and bias vector for gate x, respectively.

**Fig 4:**
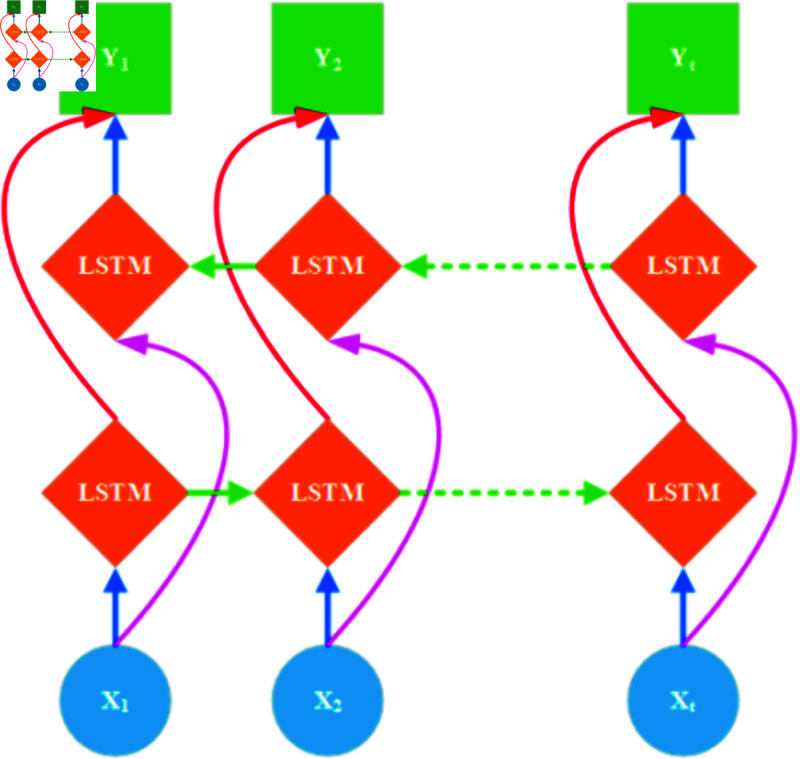
Bi-LSTM architecture diagram.

The Bi-LSTM network structure comprises a forward and backward LSTM [46]. As illustrated in Fig 4, this architecture diagram clearly shows the Bi-LSTM’s structure. By introducing the bidirectional information propagation mechanism and leveraging the advantages of LSTM units, BiLSTM demonstrates significant advantages in multiple aspects.

Firstly, by considering both the forward and backward information of the input sequence simultaneously, BiLSTM can capture context features in sequences more comprehensively. This bidirectional information propagation mechanism enables BiLSTM to more accurately understand the semantics and grammatical structure of sentences when processing sequence data. Secondly, due to the gating mechanisms and cell states introduced within LSTM units, BiLSTM can effectively mitigate the issues of gradient vanishing and exploding gradients faced by traditional RNNs when processing long sequences. Therefore, BiLSTM is capable of capturing long-term dependencies in sequences, which is crucial for handling complex sequence data. Lastly, BiLSTM can be combined with other deep learning models to form more complex hybrid models. This flexibility and scalability allow BiLSTM to adapt to various complex task requirements and demonstrate robust performance in practical applications. Due to the large fluctuation of residual components, a model with strong learning ability is required to better fit the data. Therefore, in this study, AttBiLSTM is used to predict the residual components decomposed by EMD.

### 3.7 Process of pig price prediction strategy based on STL-PCA and BWO-BiLSTM

The pig price forecasting strategy process involves the following steps:

(1) Decompose the original pig price data using STL to separate it into trend, seasonal, and residual components.
(2) Establish LSTM models for the trend and residual components to predict their trends, and develop a SARIMA model for the seasonal component to forecast its pattern.
(3) Introduce factors influencing pig prices and apply Spearman’s correlation analysis to confirm their correlation with pig prices.
(4) Use PCA to reduce the dimensionality of these factors, setting a cumulative contribution rate of 90% to obtain principal component scores.
(5) Optimize BiLSTM hyperparameters using the BWO algorithm by setting the beluga whale population size and maximum iterations, initializing population positions randomly, and defining fitness values as the BiLSTM prediction loss function.
(6) Update the positions of the whales according to their stage, determined by calculating the equilibrium factor B using Eq 4; if *B*_*f*_>0.5, the whales are in the exploration phase and the positions are updated via Eq 5; if $B_{f}\leq 0.5$, the whales are in the exploitation phase and positions are updated via Eq 6. New positions are evaluated, ranked, and the current optimal solution is identified.
(7) Calculate the whale fall probability *W*_*f*_; if $W_{f}>B_{f}$, update positions according to Eq 8.
(8) Check if the current iteration reaches the maximum limit; if so, terminate and output the optimal parameters and results; otherwise, return to step (7) for a new search cycle.
(9) Construct a BiLSTM model using BWO-optimized parameters, inputting STL-decomposed components and PCA-reduced principal components to predict pig prices.

**Fig 5:**
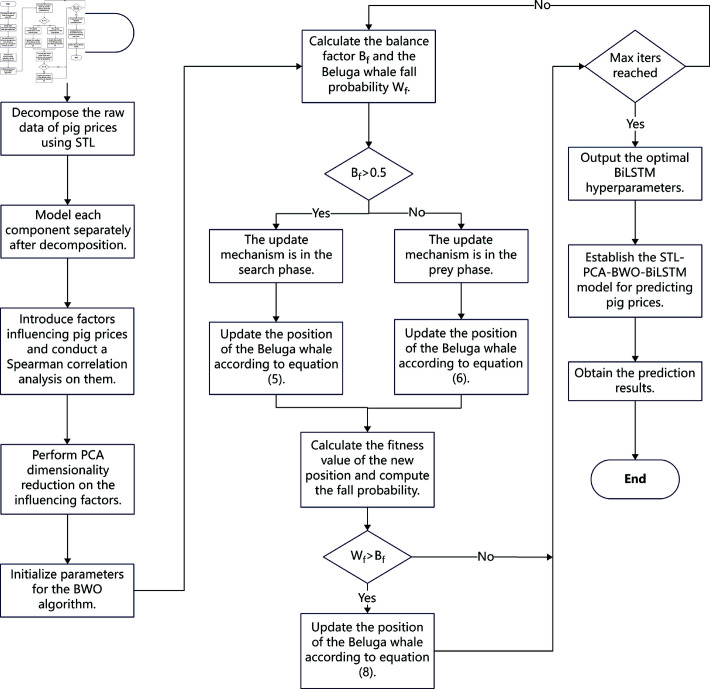
Flowchart of the STL-PCA-BWO-BiLSTM model.

The experimental flowchart based on STL-PCA and BWO-BiLSTM is shown in Fig 5:

## 4 Research design

### 4.1 Measurement criteria

In this study, we used four performance indices to measure the prediction performance of the model, root mean square error (RMSE), mean absolute error (MAE) and mean absolute percentage error (MAPE).

When the RMSE (Root Mean Squared Error) is small, it indicates that the difference between the predicted values of the model and the actual observed values is small, which means that the model has a good degree of fit. The RMSE calculation formula is as follows:

$$RMSE=\sqrt{\frac{1}{n}\sum_{i=1}^{n}{\left(Y_{i}-{\hat{Y}}_{i}\right)}^{2}}$$ (17)

MAE (Mean Absolute Error) represents the average of the absolute errors between the predicted values and the observed values. Generally, a smaller MAE value indicates that the model performs more consistently across different datasets, indicating better stability of the model. The formula for calculating MAE is as follows:

$$MAE=\frac{1}{n}\sum_{i=1}^{n}\left|Y_{i}-{\hat{Y}}_{i}\right|$$ (18)

MAPE (Mean Absolute Percentage Error) is a commonly used metric for measuring the magnitude of error between predicted values and actual values. It is the ratio of the mean absolute error of the prediction errors to the mean of the actual values, typically expressed as a percentage. A smaller MAPE value indicates that the calculated values are closer to the actual values, making the results more reliable. The formula for calculating MAPE is as follows:

$$MAPE=\frac{100\%}{n}\sum_{i=1}^{n}\left|\frac{Y_{i}-{\hat{Y}}_{i}}{Y_{i}}\right|$$ (19)

${\text{R}}^{2}$ is a statistic used to measure the goodness of fit of a regression model, with values ranging between 0 and 1. The closer it is to 1, the better the fit of the model. The formula for calculating ${\text{R}}^{2}$ is as follows:

$$R^{2}=\frac{\sum {\left({\hat{Y}}_{t}-{\bar{Y}}_{t}\right)}^{2}}{\sum {\left(Y_{t}-{\bar{Y}}_{t}\right)}^{2}}$$ (20)

In Equations (17-20), *Y*_*i*_ represents the true value of the i-th sample in the prediction data, ${\hat{Y}}_{i}$ represents the predicted value of the i-th sample in the prediction data, ${\bar{Y}}_{t}$ represents the average of the true values and n represents the total number of prediction data samples.

### 4.2 Experimental details

#### 4.2.1 Raw data prediction.

**Fig 6:**
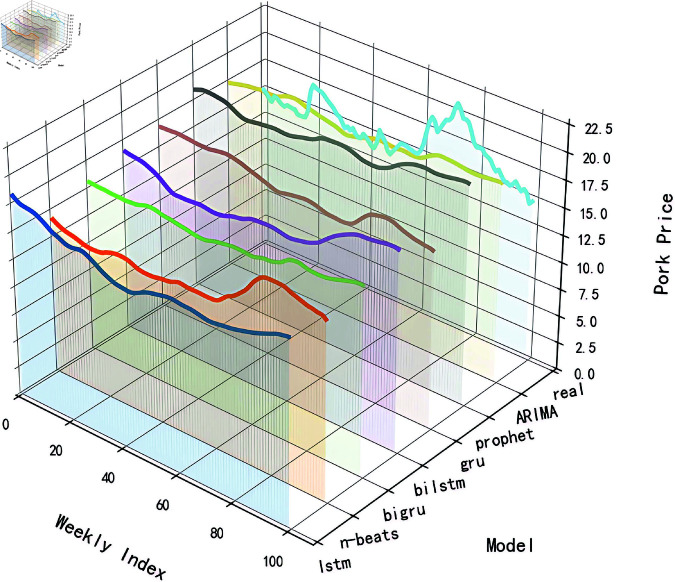
The prediction results of each model based on the original data.

To evaluate the performance of different models in predicting raw data and provide references for subsequent algorithm optimization, this study employed traditional time series algorithms including ARIMA and Prophet models, while also applying neural network models such as LSTM, GRU, Attention-based Long Short-Term Memory (AttLSTM), AttGRU, and the N-Beats model to analyze raw price data. All experimental code was implemented using Python’s TensorFlow framework. As detailed in Fig 6, while the prediction results of these models could partially reflect the trends and fluctuations of actual hog prices, their predictive performance proved unsatisfactory when the price data exhibited sharp jumps. This limitation primarily stems from the excessive magnitude of data variations, which exceeded the models’ capacity to capture both the overarching trends and nuanced patterns in price fluctuations. The Mean Absolute Error (MAE) for each model was calculated and summarized in Table 2. A lower MAE value typically indicates a smaller average deviation between the model’s predictions and the actual values, suggesting a higher degree of model fit. From the table, it can be observed that the models incorporating attention mechanisms—AttLSTM, AttGRU, and N-Beats—performed relatively better. However, none of the models were able to accurately predict the raw data.

**Table 2:** The MAE value of the model under raw data.

	Prophet	ARIMA	LSTM	GRU	N-Beats	BiLSTM	BiGRU
**MAE**	**3.09**	**2.40**	**1.71**	**1.70**	**1.67**	**1.65**	**1.32**

#### 4.2.2 STL decomposition.

**Fig 7:**
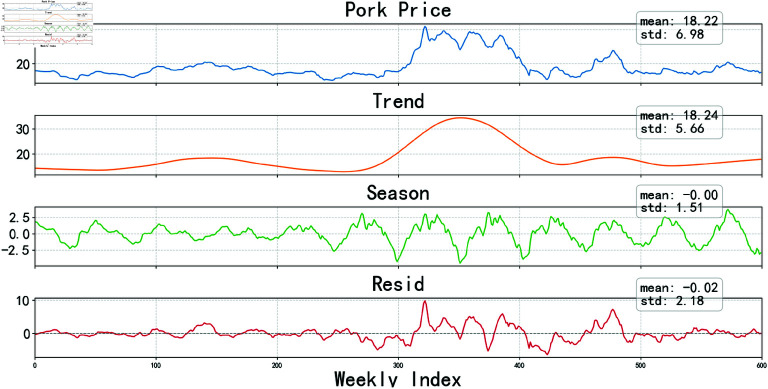
Decomposition of time scries plot.

Considering that directly predicting pork prices is difficult to achieve ideal results, this study adopts the STL model to decompose pork prices. The STL model can decompose time series data into trend, seasonal, and remainder components, which helps reduce the interactions between different data components. A detailed illustration of STL time series decomposition is provided in Fig 7.

To accurately reflect the periodicity of the original time series, the number of cycles in the time series decomposed by the STL model must be consistent with the number of cycles in the original data. Therefore, the periodic parameter of the STL model is chosen to be set at 52. Upon observing Fig 7, it can be discerned that the trend components obtained after decomposing the original time series data are smoother and exhibit lesser fluctuations compared to the original, providing a more intuitive understanding of the trend in hog price variations. Notably, the trend components consistently fluctuate up and down, without a sustained increase or decrease. The seasonal component manifests as a sequence with a periodicity of 52, while the residual component demonstrates significant short-term fluctuations and unevenness, exhibiting strong nonlinear characteristics.

To capture these complex patterns, this study employed two different modeling strategies. LSTM, a deep learning model suitable for sequential data, was used to simulate the nonlinear relationships and complex dynamics of trend and residual components. The flexibility of the LSTM model makes it an ideal choice for capturing long-term dependencies in the data. Additionally, the SARIMA model was selected to model the seasonal variables. By combining autoregressive (AR) and moving average (MA) components, the SARIMA model enables precise capture of the periodic fluctuation characteristics of seasonal time series.

**Fig 8:**
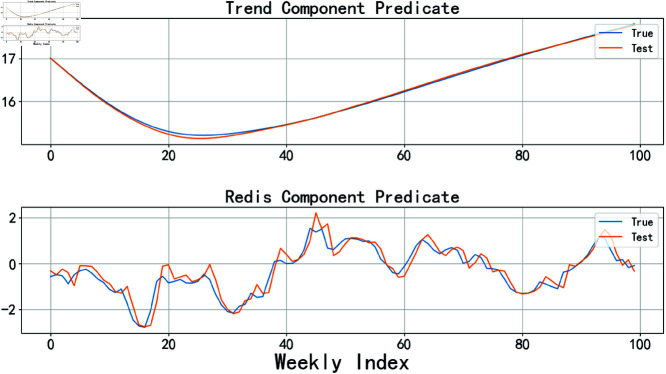
LSTM predicts trend components and residual components.

**Fig 9:**
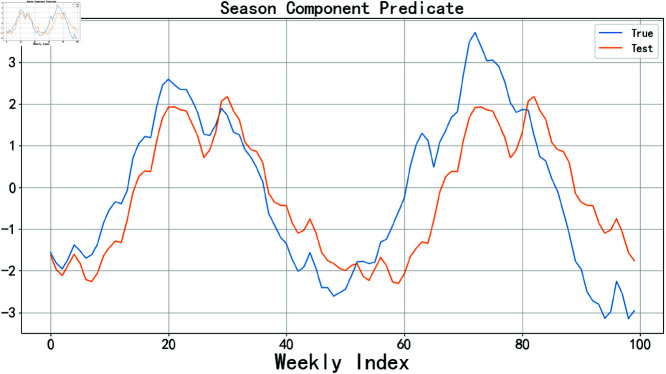
SARIMA predicts season components.

In this experiment, the experimental data was divided into two parts: 70% was used as the training set for building and optimizing the model, while the remaining 30% was used as the test set to validate the model’s performance. The prediction results for the trend component and the residual component are referenced in Fig 8, while the prediction results for the seasonal component are referenced in Fig 9.

In the ensemble algorithm of the traditional STL model, the seasonal component *S*_*t*_, trend component *C*_*t*_, and residual component *R*_*t*_ obtained from the STL model are fed into different machine learning models for training, with the training results being treated as linearly related. The results of each model training are summed using a linear combination method to obtain the final prediction result, as shown in Eq 21:

$${\hat{y}}_{t}={\hat{C}}_{t}+{\hat{S}}_{t}+{\hat{R}}_{t}$$ (21)

**Fig 10:**
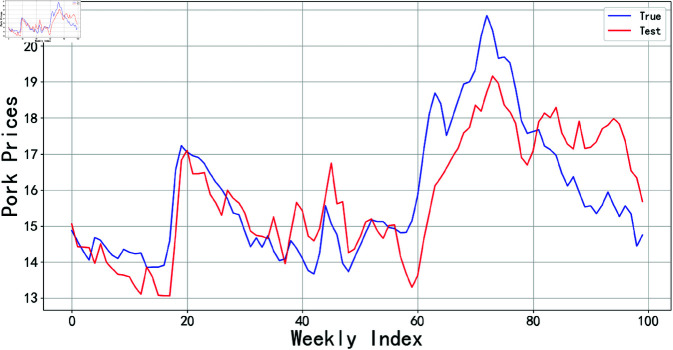
STL Linear Combination Forecast Graph.

In this equation, ${\hat{C}}_{t}$, ${\hat{S}}_{t}$, and ${\hat{R}}_{t}$ represent the trend component *C*_*t*_, seasonal component *S*_*t*_, and residual component *R*_*t*_, respectively, obtained through machine learning model training. ${\hat{y}}_{t}$ denotes the model’s prediction result after linear summation of these components. However, in practical applications, the relationships obtained through training may be both linear and nonlinear. Additionally, the linear summation method only focuses on single-column data without considering other influencing factors that may lead to lag issues. Therefore, simple linear summation may not yield ideal prediction results. The results of pig price prediction using the traditional linear summation method are shown in Fig 10. The figure indicates that the traditional linear summation method exhibits poor fitting performance during periods of significant price fluctuations and also has a lag issue.

**Fig 11:**
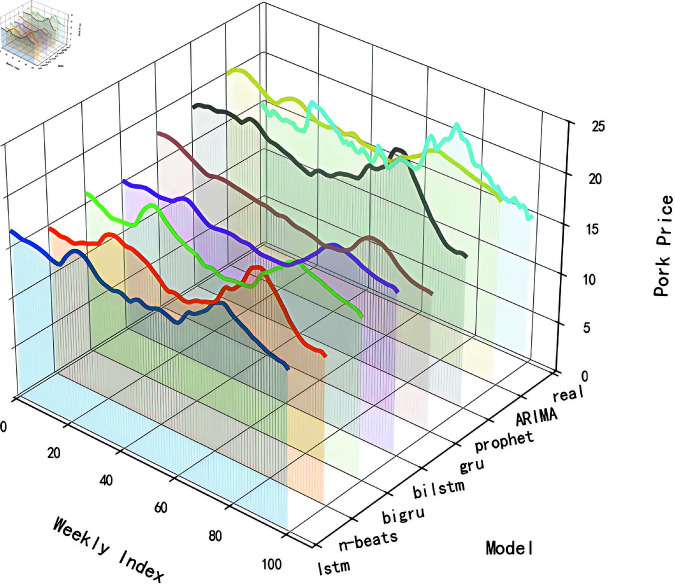
Prediction effect of each model after STL.

To investigate the effectiveness of STL decomposition in enhancing prediction performance, we compared the performance of models trained on STL-decomposed data across different algorithms. Fig 11 illustrates the prediction outcomes of various models under STL decomposition, while Fig 3 provides a quantitative comparison of MAE values between predictions derived from raw data and those utilizing STL-decomposed components. The systematic comparison of MAE metrics enables a rigorous quantification of STL decomposition’s contribution to improving model predictive capabilities.

As can be seen from Table 3, after applying STL decomposition, the MAE values of the models generally decrease, indicating an improvement in prediction accuracy. This suggests that STL decomposition technology can effectively extract and utilize the seasonal and trend information in the data, thereby enhancing prediction accuracy. However, when dealing with complex price fluctuations, the prediction capability of the models still needs further enhancement. Specifically, during periods of price volatility, the prediction results exhibit a certain degree of lag, and the errors remain significantly larger compared to when the data changes are stable. Therefore, it is necessary to further improve the prediction models to enhance their prediction performance during complex price fluctuations.

**Table 3:** The MAE value of the model after STL decomposition.

	Prophet	ARIMA	LSTM	GRU	N-Beats	BiLSTM	BiGRU	ADD
**Raw Data**	3.09	2.40	1.71	1.70	1.67	1.65	1.32	-
**STL**	2.82	3.66	1.32	1.53	0.95	1.17	1.26	0.94

#### 4.2.3 Multivariate forecasting.

Given the limitations of raw data analysis in predicting price jumps and fluctuations, as well as the potential lag issues associated with STL decomposition, this paper incorporates influencing factors based on STL-decomposed data to enhance the model’s prediction performance.

To investigate the mutual influence among different factors and their correlation with pig prices, a correlation coefficient analysis of the data is necessary. According to Fig 1, most of the influencing factors and pig prices do not conform to a normal distribution, which violates the statistical assumptions for Pearson correlation calculations. Therefore, this paper uses Spearman’s rank correlation coefficient for the analysis. Spearman’s rank correlation test is a non-parametric statistical method used to assess the correlation between the ranks of two variables. It does not require the data to follow a normal distribution and is suitable for continuous or ordinal categorical variables. The Spearman test measures the correlation by converting the original data into ranked data and then calculating the Pearson correlation coefficient between the two sets of ranked data. If the ranks of the two variables are completely consistent, the Spearman correlation coefficient is +1; if they are completely opposite, it is -1; and if there is no correlation between the variables, it is 0.

**Fig 12:**
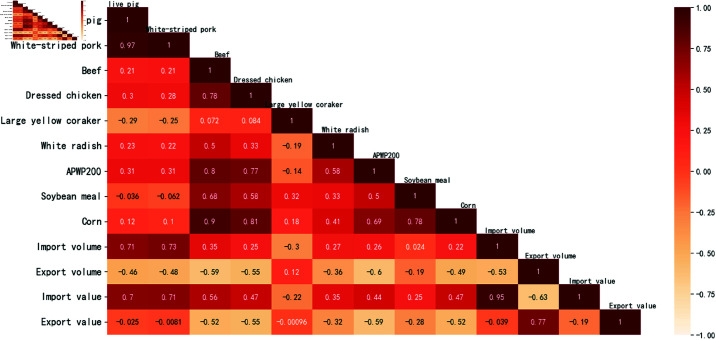
Degree of correlation between data related to live pig futures prices.

The Spearman heatmap presented in Fig 12, which displays pig prices and related factors, offers a clear insight into the correlations between different data points. However, the results indicate that only a few factors among many exhibit strong correlations, while most factors demonstrate weak correlations. To mitigate the potential interference of these weakly correlated data on the experimental results while ensuring data integrity, this study employs data dimensionality reduction to address this issue. Dimensionality reduction techniques can transform high-dimensional datasets into lower-dimensional representations, aiding in better understanding and effective prediction of the data. In Principal Component Analysis (PCA), the contribution rate and cumulative contribution rate are crucial metrics used to measure the contribution of each principal component to the original data and the cumulative contribution of the first n principal components to the original data, respectively. This represents the proportion of the total information in the original data that is retained after dimensionality reduction. Typically, if the cumulative contribution rate can reach above 80%, a relatively large amount of information will be preserved. In this paper, a contribution rate of 90% is selected to ensure sufficient information retention. The cumulative contribution rate for dimensionality reduction using PCA on pig price-related data is shown in Table 4. By extracting 4 principal components, the requirement of a 90% cumulative contribution rate is met.

**Table 4:** Accumulated contribution rate of PCA.

Number of PCA	1	2	3	4
cumulative explained variance	53.37%	78.90%	85.49%	90.22%

**Fig 13:**
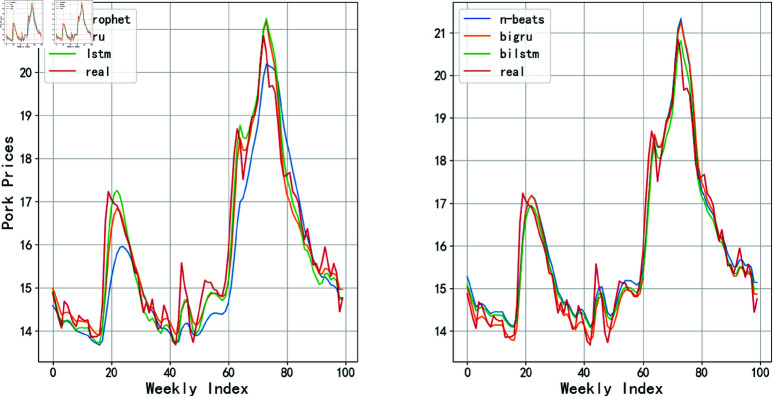
Model prediction results with added influencing factors after STL decomposition.

After applying the PCA method for dimensionality reduction, the reduced-dimensionality data and the data decomposed by STL are jointly input into the model for training. The multivariate prediction results after dimensionality reduction are shown in Fig 13, and the comparison of Mean Absolute Error (MAE) between the original prediction results, STL prediction results, and the reduced-dimensionality prediction results is presented in Table 5. The prediction curves after dimensionality reduction are closer to the actual data points, and the lag issue is also resolved. Especially in the regions where data fluctuations are large, the reduced-dimensionality model demonstrates better fitting capability. This indicates that dimensionality reduction not only simplifies the data structure but also improves the prediction performance of the model to a certain extent.

**Table 5:** MAE values under STL model and PCA principal component analysis model.

	Prophet	ARIMA	LSTM	GRU	N-Beats	BiLSTM	BiGRU
Raw Data	3.09	2.40	1.71	1.70	1.67	1.65	1.32
STL	2.82	3.66	1.32	1.53	0.95	1.17	1.26
STL-PCA	0.55	-	0.33	0.30	0.32	0.27	0.28

After comparing the MAE values and fitting curves of different models, it was found that the performance of the BiLSTM models was significantly better than other models. Based on this, this study will improve upon the BiLSTM models to achieve better prediction results.

#### 4.2.4 BiLSTM combined model.

LSTM possesses powerful dynamic modeling capabilities, capable of capturing long-term dependencies in time series data through memory cells and gating mechanisms. Therefore, it is widely used in prediction tasks. However, LSTM faces issues such as the difficulty in determining its model’s hyperparameters and weak generalization ability, which significantly impact the prediction accuracy of the predictive model. To address these issues, intelligent optimization algorithms can be utilized to optimize the hyperparameters of the LSTM prediction model.

In order to select an appropriate optimization algorithm for tuning the hyperparameters of BiLSTM, we combine the Genetic Algorithm (GA), Particle Swarm Optimization (PSO), Dung Beetle Optimizer (DBO), Whale Optimization Algorithm (WOA), and Beluga whale optimization (BWO) with the BiLSTM model to establish prediction models. Firstly, the models are trained using the original pig price data. To ensure the scientificity and fairness of the experimental results, the population size for all optimization algorithms is set to 40, the maximum number of iterations is set to 50, and the MSE of the test set is used as the fitness function for the optimization algorithms. For the BiLSTM model, the number of hidden layer units is set to 100, the maximum number of training epochs is set to 100, the initial learning rate is set to 0.001, and the L1 regularization parameter is set to 0.0001. The search ranges for the BiLSTM network parameter settings obtained by each optimization algorithm are as follows: number of hidden layer units [50, 200], maximum number of training epochs [50, 200], initial learning rate [0.0001, 0.3], and L1 regularization parameter [0.00001, 1]. The optimization results of each algorithm are shown in Table 6. The error results of the test set are shown in Table 7.

**Table 6:** Parameter optimization results of BiLSTM.

Algorithm	Hidden layer units	Training epochs	Learning rate	L1 regularization parameter
GA	126	141	0.089	0.588
PSO	82	113	0.100	0.229
DBO	86	126	0.090	0.087
WOA	164	136	0.071	0.105
BWO	163	150	0.012	0.00001

**Table 7:** Testing set error.

	BiLSTM	GA-BiLSTM	PSO-BiLSTM	DBO-BiLSTM	WOA-BiLSTM	BWO-BiLSTM
**RMSE**	2.01	3.45	2.59	2.02	1.96	1.44
**MAE**	1.65	3.06	2.39	1.70	1.62	1.22
**MAPE**	10.15%	20.59%	15.86%	10.78%	9.92%	7.97%

**Fig 14:**
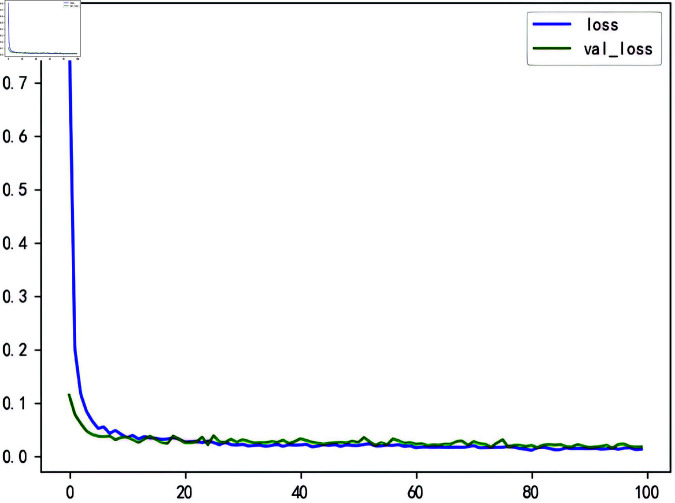
LOSS curves for BWO-BiLSTM model training.

**Fig 15:**
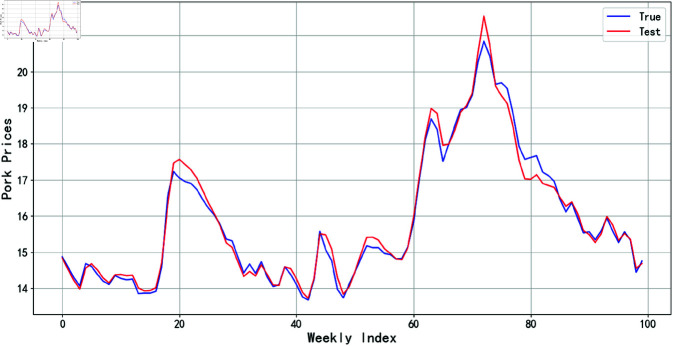
BWO-BiLSTM model prediction results.

As shown in Table 7, the three evaluation metrics of BWO-BiLSTM are all the minimum values among all models. Therefore, this study ultimately employs BWO to optimize the parameters of BiLSTM, using the hog prices combined with the data decomposed by STL and the influencing factors reduced by PCA as model inputs to predict the trend of hog prices. In Fig 14, we can observe the LOSS curve for the AttBiGRU model training. From this, we can discern the rationality of the model’s design and the effectiveness of parameter tuning, which enable the model to thoroughly learn the characteristics of the data during the training process and generalize well to unseen data. Consequently, this model does not suffer from the issue of “overfitting.” The prediction results are shown in Fig 15.

## 5 Results and discussions

In this paper, multiple forecasting models are constructed and compared, including ARIMA, Prophet, LSTM, GRU, BiLSTM, BiGRU, and N-Beats, among others. Below, we utilize RMSE, MAE, MAPE, and R2 to test whether the proposed methods can significantly enhance the accuracy of pork price forecasting. These metrics collectively reflect the accuracy, stability, and consistency of the model’s forecasting results.

The comparative experiments are divided into two phases: The first phase involves raw data forecasting, where the models are directly trained and used for forecasting with raw data to assess their baseline performance. The second phase incorporates decomposition techniques and data dimensionality reduction for model forecasting. In this phase, the STL time series decomposition technique is employed to preprocess the raw data, while PCA is used to reduce the dimensionality of influencing factors. Through comparative experiments in these two phases, we can comprehensively evaluate the performance of each forecasting model under different data processing conditions. The results are presented in Tables 8 and 9.

**Table 8:** Analysis of raw data prediction results.

	Prophet	ARIMA	LSTM	GRU	N-Beats	BiLSTM	BiGRU
**RMSE**	3.45	2.60	2.22	2.02	2.05	2.01	1.60
**MAE**	3.09	2.40	1.71	1.70	1.67	1.65	1.32
**MAPE**	20.60%	15.86%	10.56%	10.78%	10.13%	10.15%	8.13%
$R^{2}$	-2.74	-1.12	-0.54	-0.28	-0.32	-0.27	0.20

**Table 9:** Analysis of STL-PCA prediction results.

	Prophet	LSTM	GRU	N-Beats	BiLSTM	BiGRU	BWO-BiLSTM
**RMSE**	0.78	0.44	0.45	0.42	0.39	0.41	0.22
**MAE**	0.55	0.33	0.30	0.32	0.27	0.28	0.16
**MAPE**	3.32%	1.99%	1.87%	2.04%	1.70%	1.72%	0.99%
$R^{2}$	0.81	0.93	0.94	0.94	0.96	0.95	0.98

When using raw data, the BiGRU model exhibits the smallest prediction error, with RMSE, MAE, and MAPE values of 1.60, 1.32, and 8.13%, respectively. After applying STL time series decomposition and PCA dimensionality reduction to the data, the BiLSTM model performs even better, with RMSE, MAE, and MAPE values reduced to 0.39, 0.27, and 1.7%. Based on this, the paper further optimizes the BiLSTM using BWO to achieve the best performance, yielding RMSE, MAE, and MAPE values of 0.22, 0.16, and 0.99%. Moreover, it can be observed that the application of STL-PCA significantly enhances the prediction performance of the model, surpassing the previously mentioned traditional linear combination prediction method based on STL decomposition of time series data.

**Fig 16:**
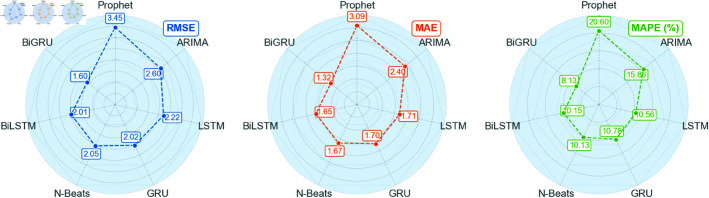
Three error values under raw data.

**Fig 17:**
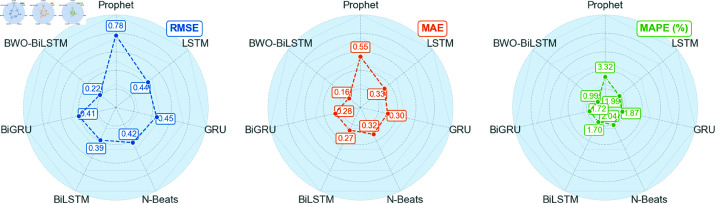
Three error values under STL decomposition and factor analysis.

A radar chart can clearly demonstrate the performance of various models across different evaluation metrics, facilitating intuitive comparisons among metrics such as RMSE, MAE, MAPE, and others. In this paper, radar charts are employed for visualization, which aids in more intuitively presenting the differences among models in terms of key evaluation metrics, as shown in Figs 16 and 17.

**Fig 18:**
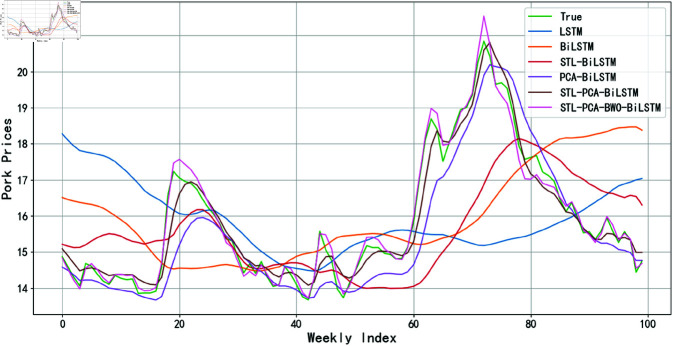
Figure of ablation experiment.

An ablation experiment is conducted to investigate the effect of incremental improvements on the prediction accuracy of the model. Starting with a basic LSTM model, a bidirectional structure, BiLSTM, is introduced. Subsequently, by incorporating STL, the STL-BiLSTM model is constructed. To further enhance the model’s performance, PCA is fused to obtain the STL-PCA-BiLSTM model. On this basis, by integrating the BWO optimization algorithm, the final STL-PCA-BWOBiLSTM model is formed. To clearly demonstrate the effectiveness of these improvements, the prediction results of the model at each stage are compared with actual pig prices, as shown in Fig 18.

## 6 Conclusions

In recent years, due to the impact of multiple factors such as the novel coronavirus, wars, and the global economic situation, the prices of agricultural products such as meat and grain in China have frequently experienced significant fluctuations. The prices of agricultural products are influenced by various factors and exhibit irregular fluctuations characterized by non-stationarity and nonlinearity, posing significant challenges to the stable development of the agricultural product market. Accurately predicting the trends of agricultural product price changes can help relevant merchants stay informed about market trends and formulate production and sales plans in a timely and accurate manner to achieve maximum industrial benefits. At the same time, it can also provide data support and decision-making basis for the government to regulate the market and introduce policies, thereby promoting the healthy and stable development of the agricultural industry.

To successfully predict agricultural product price data, this paper innovatively proposes an STL-PCA-BWO-BiLSTM prediction model, which is specifically designed to address the limitation of traditional neural network models in predicting agricultural product prices during periods of significant fluctuations. The model employs STL time series decomposition technology to break down the original price data into trend, seasonal, and residual components, each of which is precisely predicted. By integrating PCA, the model achieves data dimensionality reduction and feature extraction, thereby enhancing computational efficiency and prediction accuracy. Additionally, the BWO algorithm is utilized to intelligently optimize the model parameters, further improving the model’s application effectiveness. Through empirical research, this paper validates the superior performance of the model in predicting pork prices, providing new theoretical support and practical guidance for agricultural product price prediction.

Although this study has achieved satisfactory prediction results, there are still some limitations. Firstly, when predicting future trends in hog prices, only significant influencing factors and historical hog prices were considered. If more factors that have a significant impact on hog price fluctuations could be included in the optimization of the prediction model, the prediction accuracy could be improved to some extent. Secondly, the lag effects of influencing factors, especially those related to feed, were not taken into account. These factors often have a lagging influence on pork prices. Therefore, this study still has considerable room for development and improvement, and further in-depth research is needed. In summary, based on the work of this study, there are still many challenges to be addressed in the future. Only by continuously exploring various aspects of the agricultural product market and refining and optimizing models to improve prediction accuracy can we more accurately predict agricultural product price trends and provide clearer and more effective guidance for related industries.
